# Facebook Apps for Smoking Cessation: A Review of Content and Adherence to Evidence-Based Guidelines

**DOI:** 10.2196/jmir.3491

**Published:** 2014-09-09

**Authors:** Megan A Jacobs, Caroline O Cobb, Lorien Abroms, Amanda L Graham

**Affiliations:** ^1^Schroeder Institute for Tobacco Research and Policy Studies, LegacyWashington, DCUnited States; ^2^The Milken Institute School of Public HealthThe George Washington UniversityWashington, DCUnited States; ^3^Department of OncologyGeorgetown University Medical Center / Cancer Prevention and Control ProgramLombardi Comprehensive Cancer CenterWashington, DCUnited States

**Keywords:** Facebook, smoking cessation/methods, social media, apps

## Abstract

**Background:**

Facebook is the most popular social network site, with over 1 billion users globally. There are millions of apps available within Facebook, many of which address health and health behavior change. Facebook may represent a promising channel to reach smokers with cessation interventions via apps. To date, there have been no published reports about Facebook apps for smoking cessation.

**Objective:**

The purpose of this study was to review the features and functionality of Facebook apps for smoking cessation and to determine the extent to which they adhere to evidence-based guidelines for tobacco dependence treatment.

**Methods:**

In August 2013, we searched Facebook and three top Internet search engines using smoking cessation keywords to identify relevant Facebook apps. Resultant apps were screened for eligibility (smoking cessation-related, English language, and functioning). Eligible apps were reviewed by 2 independent coders using a standardized coding scheme. Coding included content features (interactive, informational, and social) and adherence to an established 20-item index (possible score 0-40) derived from the US Public Health Service’s Clinical Practice Guidelines for Treating Tobacco Use and Dependence.

**Results:**

We screened 22 apps for eligibility; of these, 12 underwent full coding. Only 9 apps were available on Facebook. Facebook apps fell into three broad categories: public pledge to quit (n=3), quit-date–based calculator/tracker (n=4), or a multicomponent quit smoking program (n=2). All apps incorporated interactive, informational, and social features except for two quit-date–based calculator/trackers apps (lacked informational component). All apps allowed app-related posting within Facebook (ie, on self/other Facebook profile), and four had a within-app “community” feature to enable app users to communicate with each other. Adherence index summary scores among Facebook apps were low overall (mean 15.1, SD 7.8, range 7-30), with multicomponent apps scoring the highest.

**Conclusions:**

There are few smoking cessation apps available within Facebook. Among those available, adherence to cessation treatment guidelines was low. Smoking cessation interventions provided via the Facebook platform are a unique and as yet untapped treatment strategy that can harness existing social support and social networks for quitting. Research is needed to examine whether apps that adhere to clinical practice guidelines for tobacco dependence treatment are more effective in promoting cessation than those that do not.

##  Introduction

Facebook use has become nearly universal and continues to increase. As of December 31, 2013, the social network site has over 1 billion global users [1]. In 2013, 57% of all American adults and 73% of those aged 12-17 years used Facebook [2]. Intensity of use is also escalating: in 2014, 64% of Facebook users visited the site on a daily basis, up from 51% in 2010 [2]. A primary channel through which users interact with Facebook is through millions of third-party software applications (apps) [3]. Facebook apps are available on personal computers and some are also accessible on mobile and/or smartphones. The broad reach and intensive use of Facebook worldwide represents a potentially powerful opportunity to deliver health-related behavior change interventions.

Facebook cessation apps may represent a unique approach to treatment that can leverage the power of social network ties [4,5]. Upon installing an app, their personal profile data and social network graph are typically made available to the app. Real-time access to an individual’s social network may facilitate the provision of social support from network members and the spread of an intervention through social networks.

To date, there have been no reviews of the content or quality of Facebook cessation apps. Two reviews of mobile apps by Abroms et al [6,7] found that they were heavily downloaded but most did not adhere to clinical guidelines. Choi et al [8] found cessation mobile apps may be limited as autonomous interventions. The goals of this study were to (1) assess the availability of Facebook apps for smoking cessation, (2) describe their approach and features, and (3) examine their adherence to an index based on the US Public Health Service’s Clinical Practice Guideline for Treating Tobacco Dependence (the Guideline).

##  Methods

### Identifying Facebook Apps

Two strategies were used to ensure we located apps likely to be encountered by the typical smoker searching Facebook for cessation assistance [9,10]. We searched for apps within Facebook using the general search toolbar with “Apps/Games” selected and using the App Center general search toolbar using cessation-related keywords (eg, “smoking”, “quit”, “cessation”, “cigarette cessation”). We used these same keywords in combination with “Facebook” in search engine queries on Google, Bing, and Yahoo! Since Internet searchers typically review only the first page of search engine results [8], the first two pages of results for each keyword were reviewed for any links or mention of a Facebook app. Links that led to a Facebook App Center page, directly to an app, or to an app page were saved for eligibility review. Searches were conducted in August 2013.

To determine eligibility for full review, two Master’s level coders catalogued basic information for each app link retrieved. This approach to app screening has been used in other reviews [6,7]. To be eligible for full review, apps had to be in English and include text related to cessation either in the Facebook App Center overview or within the app.

### Coding of Apps

Eligible apps were installed using the native Facebook platform on a personal computer or the iPhone platform for mobile-only apps. Coders used apps over 3 days with at least 3 logins to ensure all features were utilized and coded. (Co-authors MAJ and ALG were involved in the development of one of the Facebook apps that was eligible for full review. Coders of this app were research staff not directly involved in app development or the associated research grant [R01 CA155369-01A1]). Apps were coded for publisher/developer type, cost, and content features (interactive, informational, and social). Operational definitions of content features were developed prior to coding and were noted as present or absent. Interactivity was defined as “any content-related user input that results in feedback from the website” [10]. Informational features were those specific to smoking cessation, withdrawal symptoms, triggers to smoke/cravings, and/or ways to deal with cravings. Social features included (1) a within-app community with communication features (eg, public posting wall, personal message function), and (2) the ability to post updates or information to a user’s Facebook wall to share information with existing social network ties. Using an existing scheme [6,7], apps were classified as public pledge (eg, choose a reason for quitting and post to Facebook wall), calculator/tracker (eg, money saved/cigarettes not smoked calculated based on quit date), or multicomponent (eg, contained a calculator/tracker feature and quitting guide).

Apps were then coded for adherence to the Guideline [11] using a modified version of an established index [6,7]. Modifications included (1) removal of “Recommend counseling and medicines” and “Refer to recommended treatment” because they duplicated other “ASSIST with a quit plan” items, (2) editing of “Text alerts” to “Notifications (any type including text)” given alternative notification options within Facebook, and (3) addition of “Recommend talking to your health care provider about quitting” as key feature of cessation treatment (Table 1). Items were scored on a 3-point scale (0=not at all present, 1=partially present, or 2=fully present). For example, for the guideline to “ARRANGE for follow-up”, apps that did not mention any follow-up or send an invitation to return to the app received a 0; apps that either mentioned follow-up or sent an invitation to return received a score of 1; and apps that did both received a score of 2. For items such as “Enhance motivation: rewards”, apps that referred to specific rewards (eg, whiter teeth) received a 2; apps that referred only to generic rewards (eg, “quitting has lots of benefits”) received a 1; and apps that did not refer to any rewards received a 0.

Similar to Abroms et al [7], when scores between coders differed by 2 points (ie, 0 vs 2), coders discussed the discrepancy and recoded the item. After recoding, if item scores differed by 1 point or less, two scores were averaged; all item scores for each app were summed (maximum adherence score possible=40).

### Data Analysis

Frequencies and descriptive statistics were used to characterize apps. For each adherence index item, the proportion of apps receiving a 2 (fully present) by at least one coder was calculated (average score≥1.5; as in [7]). The relationship between publisher/developer source and app type on the adherence index summary score was examined. All analyses were performed in SPSS Version 21.

##  Results

### Summary

**Figure 1:**
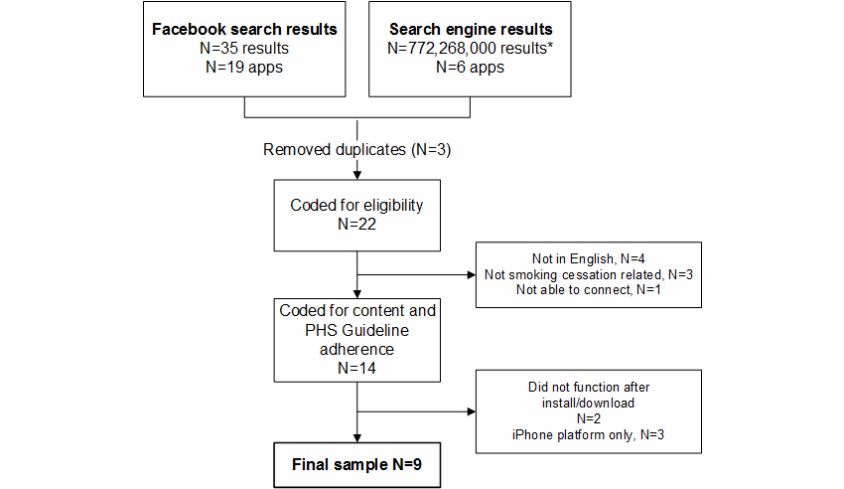
Study flow diagram from search results to final sample of apps reviewed (total number of results from Yahoo! search engine query were not available).

The search resulted in 19 apps from within Facebook’s internal search platform and 6 apps from Internet search engines (Figure 1). After removing duplicates (n=3), those available only on iPhone (n=3), and ineligible (n=8) and non-functional (n=2) apps, the final sample included 9 apps that were available on Facebook (see Multimedia Appendix 1).

### Facebook App Characteristics

Three apps were sponsored by a pharmaceutical company, three were funded/sponsored by a government entity, and three originated from an individual/private company or were unclear. As required by Facebook [12], all apps were free to install. All apps were interactive, and most contained informational features (7/9). Only four apps had a within-app “community” feature to enable app users to communicate with each other. All apps allowed app-related posting within Facebook (ie, on self/other Facebook profile). Four apps were categorized as calculator/tracker apps, three were related to a public pledge to quit, and two were coded as multicomponent (see Multimedia Appendix 2 for representative screenshots from each category).

### Adherence Index Results

Reviewer agreement was 67% or better for 17 of 20 items. Agreement for the remaining 3 items fell between 44% and 56%: “ASSIST with a quit plan: supplementary information”, “Enhance motivation: risk”, and “Enhance motivation: roadblocks”. Table 1 displays the proportion of apps that received a “fully present” rating by at least one coder (ie, average score≥1.5 for a given item).

Only 3 of 20 adherence index items were fully present in the majority of apps: “Specific to smoking”, “Enhance motivations: Rewards”, and “Interactive”. The average adherence index score was 15.1 (SD 7.8; N=9). Government sponsored/funded apps scored higher than other publisher/developer types, and multicomponent cessation apps scored higher than calculator/tracker and public pledge to quit apps (Table 2).

**Table 1:** Adherence index item endorsement across Facebook apps (N=9).

Adherence index item	n (%) of apps^a^
1. Specific to smoking	9 (100)
2. ASK for smoking status	4 (44.4)
3. ASSESS willingness to quit	2 (22.2)
4. ADVISE every user to quit	3 (33.3)
5. ADVISE every user to quit: personalized	2 (22.2)
6. ADVISE every user to quit: clear	2 (22.2)
7. ADVISE every users to quit: strong	2 (22.2)
8. ASSIST with a quit plan: overall plan	2 (22.2)
9. ASSIST with a quit plan: practical counseling	3 (33.3)
10. ASSIST with a quit plan: recommend approved meds	3 (33.3)
11. ASSIST with a quit plan: intra-treatment social support	4 (44.4)
12. ASSIST with a quit plan: supplementary information	1 (11.1)
13. ARRANGE for follow-up	1 (11.1)
14. Enhance motivation: rewards	7 (77.8)
15. Enhance motivation: risks	3 (33.3)
16. Enhance motivation: roadblocks	3 (33.3)
17. Connect to a quitline	1 (11.1)
18. Interactive	9 (100)
19. Notifications (any type including text)	1 (11.1)
20. Talk with healthcare provider re: quitting	1 (11.1)

^a^Proportion of apps with at least one coder indicating item was “fully present” (total N=9).

**Table 2:** Facebook app adherence index summary score by publisher/developer and category (N=9).

Review category		Mean (SD)^a^
**By publisher/developer**		
	Individual/other (n=3)	13.3 (9.8)
	Pharmaceutical company (n=3)	15.5 (1.3)
	Government group (n=3)	16.3 (11.9)
**By category**		
	Calculator/Tracker (n=4)	11.6 (8.7)
	Public pledge to quit (n=3)	14.5 (1.8)
	Multicomponent cessation program (n=2)	22.8 (9.5)

^a^Summary score across all adherence index items; absolute range 0-40.

## Discussion

### Principal Findings

We identified only 9 English-language Facebook smoking cessation apps. Overall, apps scored poorly on adherence to clinical recommendations for tobacco dependence treatment. Referrals to quitlines and health care providers were infrequent. Consistent with earlier reviews [6,7], treatment components completely absent from most Facebook apps included personalized advice to quit and notifications. These omissions are noteworthy given the ease with which Facebook makes it possible to personalize app experiences and send notifications.

All apps included in-app social support and social media integration. Facebook’s social environment may make it easier for apps to leverage these tools relative to other kinds of mobile app platforms. Future research should explore the use and effectiveness of app-related social support and examine the extent to which users have concerns about sharing smoking/quitting information with their Facebook network [13]. Mobile and Facebook app designs for smoking cessation or other health behavior change could benefit from knowing which components correlate with effectiveness.

### Limitations

A limitation of the study is that apps were scored using an adherence index based on clinical interventions delivered by health care providers [7], which may not be the most suitable criteria for technology-based approaches to smoking cessation. Also, the use of social networking within apps was not explored. Future app reviews should specifically consider social networking features and the unique social network elements of Facebook apps. A strength of the study was the use of apps for a minimum of 3 days by two independent coders to ensure that all content and functionality in the app were experienced, including elements that revealed themselves after several uses. Our methods add important information to other studies [14,15] that have not employed this approach.

### Conclusions

Facebook apps for cessation are few in number and generally lack core components of evidence-based cessation treatment. Research is needed to determine whether adherence to treatment guidelines is related to effectiveness in promoting cessation.

## Multimedia Appendix 1

Final sample of Facebook and iPhone apps coded.

## Multimedia Appendix 2

Representative screenshots from apps in each category.
