# Supplementary material for: Facebook Apps for Smoking Cessation: A Review of Content and Adherence to Evidence-Based Guidelines
Source: J Med Internet Res. 2014 Sep 9;16(9):e205. doi: 10.2196/jmir.3491 (PMC4180329; doi:10.2196/jmir.3491)
Supplement: Supplementary file 1 [file jmir_v16i9e205_app1.pdf]

Appendix 1. Final sample of Facebook and iPhone apps coded

Facebook apps (N=9)

Anti-Smoking Campaign

Cessation Nation

I QUIT

Nicorette 12 Week Quit Plan

Pledge to Quit

Quit O Meter

Quit With Me

UbiQUITous

What have you been smoking?

iPhone apps (N=3)

Butt Out v 1.91

Crush the Crave

Kwit
