# Supplementary material for: Facebook Apps for Smoking Cessation: A Review of Content and Adherence to Evidence-Based Guidelines
Source: J Med Internet Res. 2014 Sep 9;16(9):e205. doi: 10.2196/jmir.3491 (PMC4180329; doi:10.2196/jmir.3491)

Appendix 2. Representative screenshots from apps in each category

A. Screenshot of public pledge to quit Facebook app (I QUIT)

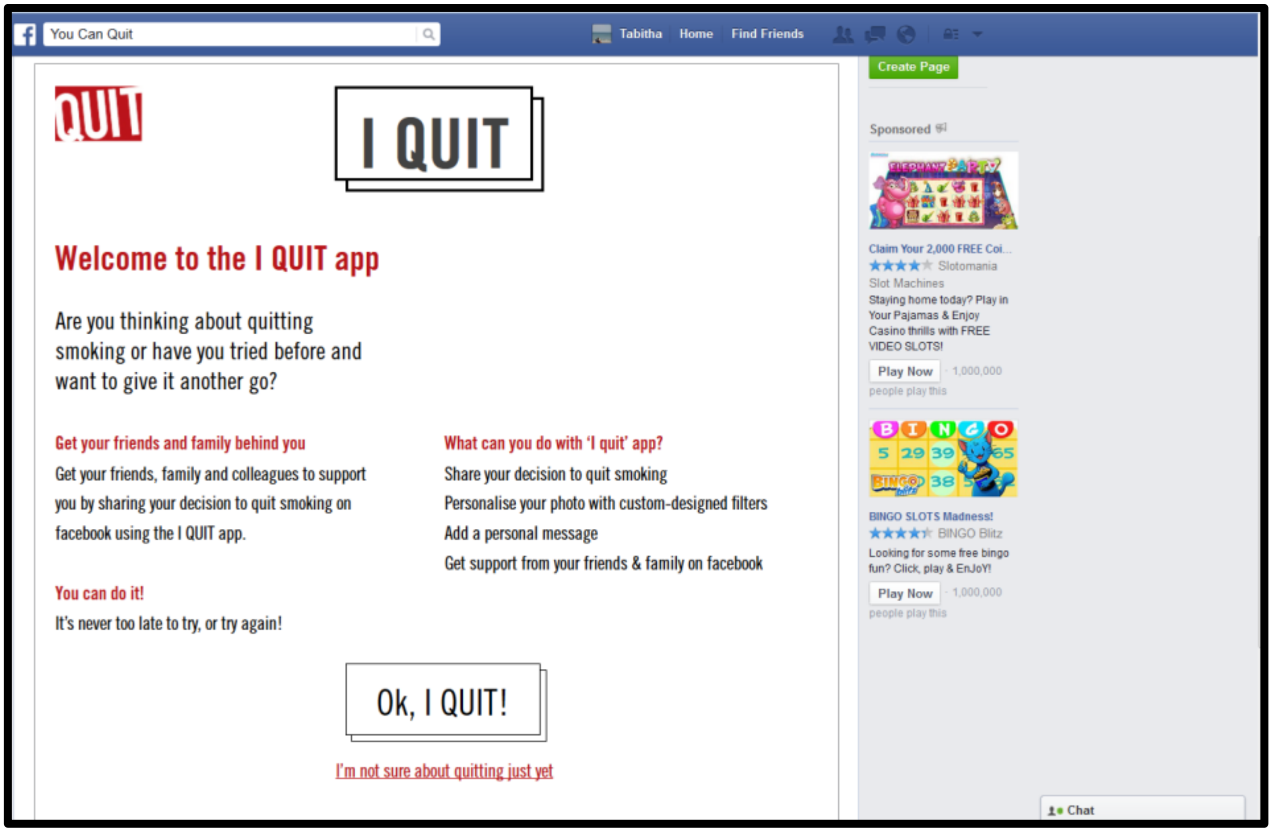

B. Screenshot of calculator/tracker Facebook app (Cessation Nation)

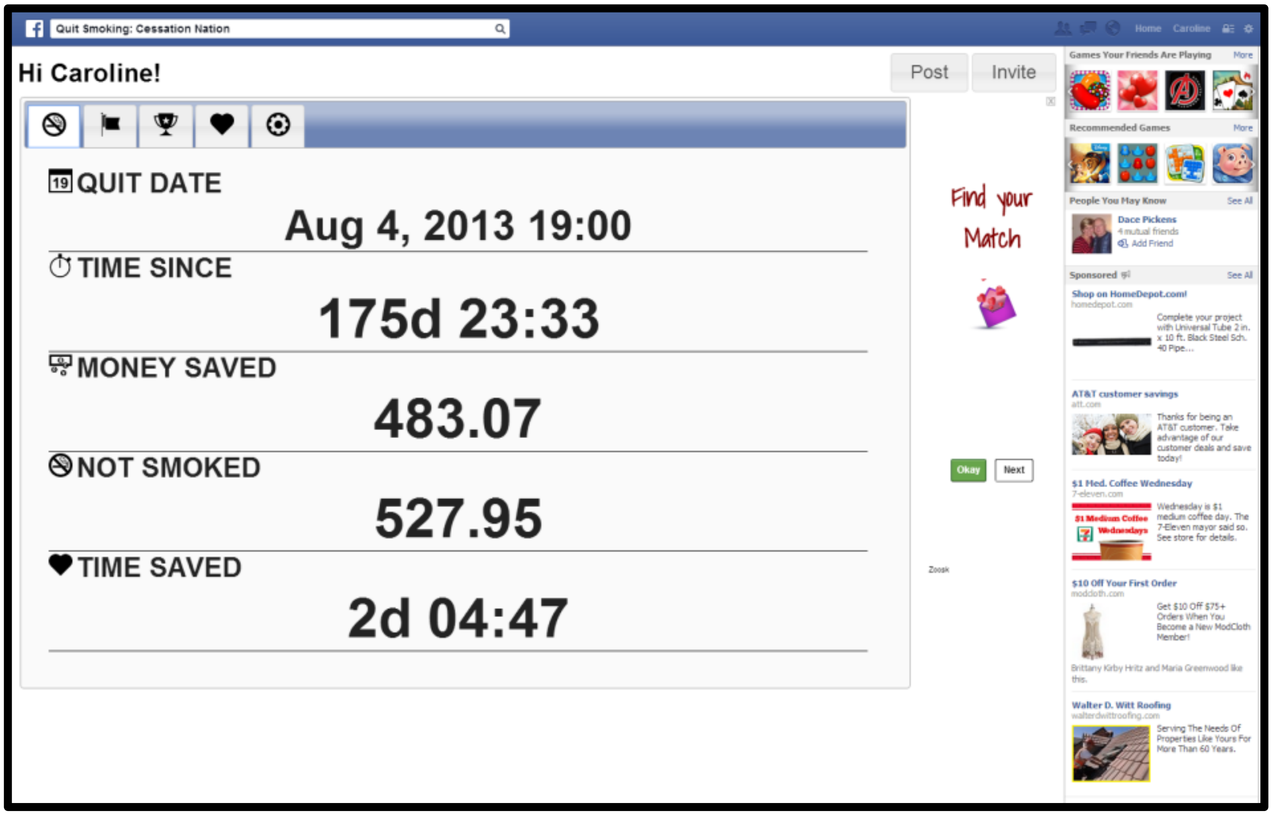

C. Screenshot of multi-component quit smoking program Facebook app (UbQUITOUS)

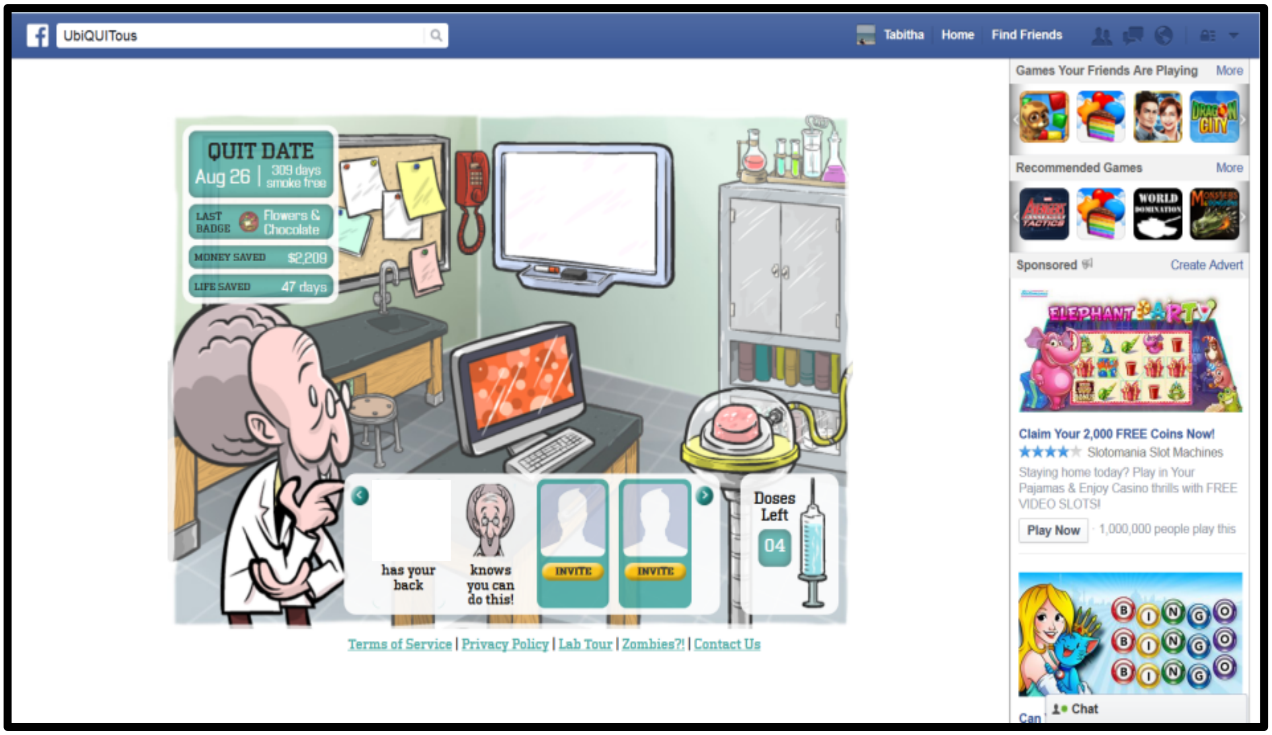

Supplement: Supplementary file 2 [file jmir_v16i9e205_app2.pdf]
